# Mental health among outpatient reproductive health care providers during the US COVID-19 epidemic

**DOI:** 10.1186/s12978-021-01102-1

**Published:** 2021-02-24

**Authors:** Alison B. Comfort, Paul J. Krezanoski, Lavanya Rao, Alison El Ayadi, Alexander C. Tsai, Suzan Goodman, Cynthia C. Harper

**Affiliations:** 1 Bixby Center for Global Reproductive Health, Department of Obstetrics, Gynecology, and Reproductive Sciences, University of California San Francisco, 550 16th Street, 3rd floor, San Francisco, CA 94143 USA; 2 Department of Medicine, University of California San Francisco, Zuckerberg San Francisco General Hospital, 1001 Potrero Ave, San Francisco, CA 94110 USA; 3 Bixby Center for Global Reproductive Health, Department of Family and Community Medicine, University of California San Francisco, 550 16th Street, 3rd floor, San Francisco, CA 94143 USA; 4 Massachusetts General Hospital, Harvard Medical School, 125 Nashua Street, Suite 722, Boston, MA 02114 USA

**Keywords:** COVID-19, Mental health, Stress, Anxiety, Outpatient providers, Reproductive health providers, Contraceptive care

## Abstract

**Introduction:**

Both inpatient and outpatient providers may be at increased risk of stress, anxiety and depression from their roles as health providers during the COVID-19 epidemic. This study explores how the US COVID-19 epidemic has increased feelings of stress, anxiety and depression among outpatient reproductive health providers.

**Methods:**

We conducted a survey with open-ended responses among outpatient reproductive health providers across the U.S. engaged in contraceptive care to collect data on their experiences with stress, anxiety and depression during the COVID-19 epidemic. The study population included physicians, nurses, social workers, and other health professions [n = 288]. Data were collected from April 21st–June 24th 2020. We used content analysis of free text responses among providers reporting increased stress, anxiety or depression.

**Results:**

Two-thirds (184) of providers reported increased stress and one-third (96) reported increased anxiety or depression related to care provision during the COVID-19 epidemic. The major sources of stress, anxiety and depression were due to patient care, worry about becoming infected or infecting family members, work- and home-related concerns, experiencing provider burnout, and fear of the unknown. Concerns about quality of patient care, providers’ changing responsibilities, lack of personal protective equipment, and difficulty coping with co-worker illness and absence all contributed to provider stress and anxiety. Worries about unemployment and childcare responsibilities were also highlighted. Providers attributed their stress, anxiety or depression to feeling overwhelmed, being unable to focus, lacking sleep, and worrying about the unknown.

**Conclusions:**

US outpatient providers are experiencing significant stress, anxiety, and depression during the US COVID-19 epidemic. Policy and programmatic responses are urgently needed to address the widespread adverse mental health consequences of this epidemic on outpatient providers, including reproductive health providers, across the US.

**Plan English summary:**

Both inpatient and outpatient providers may be at increased risk of stress, anxiety and depression from their roles as health providers during the COVID-19 epidemic. This study explores how the US COVID-19 epidemic has increased feelings of stress, anxiety and depression among outpatient reproductive health providers across the US. We conducted a survey from April 21st to June 24th, 2020 among outpatient reproductive health providers, including physicians, nurses, social workers and other health professions. We asked open-ended questions to understand why providers reported increased stress, anxiety and/or depression. Two-thirds (184) of providers reported increased stress and one-third (96) reported increased anxiety or depression from care provision during the COVID-19 epidemic. Major sources of stress, anxiety and depression were due to patient care, worry about becoming infected or infecting family members, work- and home-related concerns, experiencing provider burnout, and fear of the unknown. Concerns about quality of patient care, providers’ changing responsibilities, lack of personal protective equipment, and difficulty coping with co-worker illness and absence all contributed to provider stress and anxiety. Worries about unemployment and childcare responsibilities were also highlighted. Providers attributed their stress, anxiety or depression to feeling overwhelmed, being unable to focus, lacking sleep, and worrying about the unknown. This study highlights that US outpatient reproductive health providers are experiencing significant stress, anxiety, and depression during the US COVID-19 epidemic. Policy and programmatic responses are urgently needed to address the widespread adverse mental health consequences of this epidemic on outpatient providers, including reproductive health providers, across the US.

## Introduction

The severe coronavirus disease 2019 (COVID-19) pandemic has created significant challenges to the provision of health care in the US [1–3]. Front-line providers who treat COVID-19 patients in the emergency and inpatient settings face tremendously stressful working environments, with high patient mortality, inadequate supplies of personal protective equipment (PPE), and feelings of powerlessness and exhaustion due to patient volume. While the stress faced by front-line providers is apparent, the mental health consequences for providers, including reproductive health providers, offering care in the outpatient setting during this pandemic are less clear.

Access to comprehensive contraceptive services has become increasingly challenging during the COVID-19 pandemic. Reproductive health providers have faced significant obstacles in offering comprehensive contraceptive services during this pandemic [4, 5]. Contraceptive providers, similar to other health providers, have needed to adjust how care is provided to accommodate shelter-in-place and social distancing recommendations, respond to changes in clinic funding, and satisfy competing demands for their time for COVID-related care [5]. As a result of the changing nature of their roles in response to the pandemic, there are many ways in which these reproductive health providers may be at high risk of burnout, anxiety and depression. While there is an impending public health crisis from the mental health consequences of COVID-19 among the general population [6], there is also an urgent need to focus on the mental health implications among health care providers as well, including reproductive health providers.

There is growing interest in the implications of the epidemic on providers’ stress levels, anxiety and depression [7–11]. Recent examples in the press have highlighted that providers are feeling overwhelmed [12]:

“In a medical crisis, my job is to manage a clinical team, problem-solve and be in control. It is hard to admit that I feel vulnerable and scared…But I am admitting it because you need to know how close health care workers are to breaking.”

Emergency Medicine Physician, Arizona

Emblematic of these challenges are at least two well-publicized examples of health care providers in the US who have died by suicide during this pandemic [13–15]. While these deaths cannot be directly attributed to providing care during this pandemic, the timing and circumstance of these tragedies requires that we look further into the mental health pressures of the COVID-19 pandemic.

Due to the nature of their work even before COVID-19, health providers face increased risks of stress, burnout, anxiety and depression [16–21]. These adverse consequences have been linked to decreased effectiveness, poor quality of care, and adverse personal consequences such as substance abuse, depression, and suicide [16, 22–25]. There has been limited data, however, measuring the mental health impact of the COVID-19 pandemic on health providers. A recent study on obstetricians and gynecologists in the US highlighted the compassion fatigue and shared trauma experienced by providers as a result of their proximity to the suffering of patients during the COVID-19 pandemic [26]. In China, a high proportion of providers treating patients with COVID-19 reported symptoms of depression, anxiety, insomnia, and distress [11]. Similar patterns were documented in Italy and Canada [27, 28]. A study in the US during the first few weeks of the pandemic identified multiple sources of anxiety [29], and a recent survey among US emergency physicians found increased levels of stress, burnout and emotional exhaustion during the epidemic compared to pre-pandemic levels [30].

There is no known research on the mental health consequences of the COVID-19 epidemic among reproductive health providers in the US. In this study, we investigated reproductive health providers with a focus on understanding how changes in provider roles have impacted feelings of stress, anxiety and depression. We used open-ended questions to capture, in their own voices, providers’ experiences with care provision during this epidemic to expand our knowledge of the impacts of COVID-19 on health care in the US.

## Materials and methods

### Study sample and data collection

We conducted our study among a convenience sample of reproductive health providers engaged in contraceptive care across different practice settings throughout the US. The study sample was recruited from reproductive health providers who had participated in a Continuing Medical Education-accredited contraceptive training course conducted by the University of California San Francisco School of Medicine, Department of Obstetrics and Gynecology in the past five years. Participants included those who offer clinical care or counseling about contraception. These providers worked across different outpatient settings, though some also provided inpatient care.

An online survey was emailed through Qualtrics to study participants between April 21st to June 24th 2020 that included open-ended response items on experiences during the COVID-19 epidemic. A total of 2901 surveys were emailed to potential study participants, with 3–5 reminder emails. 288 providers consented to participate in the study and met the eligibility criteria of providing clinical care, counseling, or education about contraception (10% response rate). The response rate is a significant under-estimate because certain providers were no longer working and retired when contacted. We presented descriptive statistics of the sample (n = 288). Then we focused our analysis on the free text responses of the providers who reported experiencing stress, anxiety or depression due to their work during COVID-19 epidemic, among the larger study sample of surveyed providers reporting on how care provision has changed. This analytical sample consisted of the 187 providers of the 288 who reported experiencing stress, anxiety and/or depression. Providers were asked, “In what ways have you as a provider been affected by the COVID-19 pandemic?” with “Stress” and “Anxiety/Depression” included in response categories. If participants checked either category, they were asked an open-ended follow-up question about how COVID-19 pandemic had increased these feelings. Participants who completed the survey were entered into a drawing to win a $250 amazon giftcard. The study was approved by the UCSF Institutional Review Board.

### Statistical analysis

Data were presented from the full sample of reproductive health providers completing the survey to compare socio-demographic characteristics and calculate the prevalence of providers experiencing stress, anxiety and/or depression. We then conducted a content analysis of the free text responses among the analytical sample of those providers reporting having experienced stress, anxiety and/or depression. After inductive code generation from the open-ended responses by the first author, survey responses were coded separately by the third author. The first and third author then met to resolve any inconsistencies with coding and synthesize their assessments. Key themes arising from the data were identified and summarized the findings across the themes. Representative quotes were selected to highlight examples within each theme. The analysis focused both on the frequency of different concepts and the salience of particular responses to the overall topic of the mental health impacts of the COVID-19 epidemic. Qualitative data were analyzed in Atlas.ti.

## Results

### Descriptive statistics of sample

Among the study participants (n = 288), 187 participants (65%) reported feelings of stress, anxiety and/or depression. This sample of providers who reported increased stress, anxiety and/or depression (n = 187) included 32 (18%) physicians, 8 (4%) physician’s assistants, 53 (29%) nurse practitioners and certified nurse midwives, 34 (19%) registered nurses, 6 (3%) other nurses, 7 (4%) medical assistants, 6 (3%) health educators/social workers, 20 (11%) managers/directors, 15 administrative staff (8%), and 1 nursing/medical student (1%) (Table 1). On average, providers were 46 years old and 168 (92%) identified as female. Among this sample, 113 identified as White (62%), 14 (8%) as Black, 30 (16%) as Hispanic/Latinx, 19 (10%) as Asian/Pacific Islander, 4 (2%) as Native American, and 2 (1%) as other race/ethnicity. Practice settings varied with 83 (45%) from primary care and health departments, 58 (32%) from family planning and abortion clinics, 9 (5%) from outpatient clinics at hospitals, 35 (19%) from youth/student health center, and 23 (12%) from other practice settings. Providers spanned 25 states covering all regions: Northeast (21 [11%]), Midwest (17 [9%]), Southeast (49 [26%]), Southwest (388 [26%]), and West (52 [28%]). Chi-squared testing was conducted to assess any differences in characteristics between provider who had versus those who had not experienced increased stress, anxiety and/or depression. There were no statistically significant differences by age, gender, race/ethnicity, provider type, practice setting, or geographic region (Table 1).

**Table 1:** Summary characteristics of providers

	Full Sample (n = 288)		Sample of providers affected by feelings of stress, anxiety or depression (n = 187)		χ^2^ comparison of sample that did versus did not report increased stress/anxiety/depression
	n	%	n	%	
**Sex**, n (%)					
Female	251	94	168	92	χ^2^ = 3.10
Male	14	5	12	7	p = 0.21
Other/non-binary	2	1	2	1	
**Age** (mean ± SD)	46.4 ± 11.5		46.0 ± 11.4		t-stat = 0.92, p=0.36
**Race/ethnicity**, n (%)					
White	165	62	113	62	χ^2^ = 8.38
Black	29	11	14	8	p = 0.14
Hispanic/Latinx	40	15	30	16	
Asian/Pacific Islander	26	10	19	10	
Native American	4	2	4	2	
Other	3	1	2	1	
**Provider type**, n (%)					
Physician	44	16	32	18	χ^2^ = 16.39
Physician’s Assistant	14	5	8	4	p = 0.06
Nurse Practitioner/ Certified Nurse Midwife	82	30	53	29	
Registered Nurse	61	23	34	19	
Other Nurse	7	3	6	3	
Medical Assistant	11	4	7	4	
Health Educator/Social worker	7	3	6	3	
Manager/Director	24	9	20	11	
Administrative staff	17	6	15	8	
Student	3	1	1	1	
**Education**, n (%)					
High school, GED, technical or vocational	13	5	9	5	χ^2^ = 0.40
Two-year college degree	21	8	13	7	p = 0.98
Four-year college degree	61	23	43	24	
Graduate or professional	163	61	111	61	
Other	8	3	6	3	
**Practice setting**, n (%)					
Primary care/Heath department	123	43	83	45	χ^2^ = 1.52
Family planning/abortion clinics	97	34	58	32	p = 0.68
Youth/student clinics	52	18	35	19	
Hospital/Other	16	6	9	5	
**Region**, n (%)					
Northeast	35	12	21	11	χ^2^ = 5.2723
Midwest	26	9	17	9	p = 0.260
Southeast	90	31	49	26	
Southwest	76	26	38	26	
West	69	23	52	28	

The last column represents chi-square (for categorical) and t-tests (for continuous) comparisons between the sample that reported increased stress, anxiety and depression and the sample that did not

Most reproductive health providers reported that their clinic was currently open and providing telehealth services (109 [58%]) (Table 2). At the time of the survey, three-fourths of providers (135 [75%]) said their county was re-opening with restrictions, while one-fifth said they were still under stay-at-home/shelter-in-place orders (40 [22%]).

**Table 2:** Ways that providers have been affected by COVID-19 pandemic and clinic status

	n	%
*Sample* = *all providers (n* = *288)*		
*In what ways have you as a provider been affected by COVID-19?*		
Stress	184	66
Anxiety or depression	96	35
Fear of going to clinic or getting sick	110	40
Shift towards providing COVID-19 related care	113	41
Shift towards administrative work (adjusting protocols, time in meetings)	114	41
Shift away from sexual and reproductive health services	72	26
Absenteeism (due to own illness, family illness, school closures)	43	16
Decreased staff hours	72	26
Increased staff hours	36	13
*Sample* = *providers who were affected by stress, anxiety or depression (n* = *187)*		
*Current status of clinic (at time of survey)*		
Closed completely	3	2
Closed on-site but providing services through telehealth	35	19
Open on-site and providing services through telehealth	109	58
Open on-site but not providing services through telehealth	40	21
*Current status of stay-at-home orders (at time of survey)*		
Stay-at-home/ shelter-in-place order	40	22
Re-opening with restrictions	135	75
Completely open	5	3

Among the sample of providers, two-thirds (184 [66%]) said they felt increased stress when asked in what ways they have been affected by COVID-19 (Table 2). In addition, one-third (96 [35%]) reported the epidemic had increased feelings of anxiety/depression. Other ways that providers reported they were affected by the COVID-19 epidemic included: fear of going to clinic/getting sick (110 [40%]), shift towards COVID-19 related care (113 [41%]), shift away from their primary roles (72 [26%]), and absenteeism (due to own illness, family illness or care-giving) (43 [16%]).

Responses to the open-ended questions about increased feelings of stress and/or anxiety/depression centered on the following key themes: patient care, worry about own or family infection, financial and employment-related concerns, childcare, provider distress/burnout, and the fear of the unknown. Within each of these themes, several sub-themes emerged.

### Patient care

The most commonly reported reasons for increased stress, anxiety and depression were feelings of inadequacy around patient care and follow-up. Providers were concerned that patients would not know how to access care, that patients may be isolated during shelter-in-place, and might have no way to seek help. Reproductive health providers mentioned challenges in finding new ways to offer the same quality of care, limitations of telehealth visits, and concerns that patients were not able to access care:

“I hate for a client to not know [if] they can still come see or call us. We are here for them still.” *Clinic Manager, Family Planning Clinic, Tennessee*

Study participants were also concerned that non-COVID-19 patients were not getting the needed care during this time, due to attention focused on addressing COVID-19:

“Concern for so much focus on COVID-19 leading to worsening of other health conditions.”

*Physician Assistant, County Health Department, North Carolina*

### Sudden increase in responsibilities due to COVID-19 related care

Thirty percent of the whole sample (n = 288) had shifted service provision towards COVID-19 related care. Among the providers experienced increased stress, anxiety and/or depression (n = 187), they reported having more administrative tasks, increased hours, extra responsibilities, and being short-staffed, including due to staff illness and quarantines. A clinic manager explained that their stress was due to:

“Increased workload, little sleep, urgent changes, and ever-changing needs.”

*Clinic Manager, Family Planning Clinic, Virginia*

In addition, several providers were now responsible for new roles, such as epidemic response planning or COVID-19 testing tent management. One nurse described increased responsibilities:

“Majority of our staff are assisting at the COVID drive-thru testing centers but weekly we are testing approximately 300 people in a four-hour span. It is hot, we are worn out. Then [we] are calling all of the positive and also doing the contract tracing and mailing every person that gets tested a copy of their result, positive or negative…We no longer have volunteers assisting us…[W]e are managing our normal daily routines of answering the phone calls, rescheduling patients that were scheduled prior to COVID that have to be changed to telemedicine visits.”

*Registered Nurse, Family Planning Clinic, Louisiana*

In instances where clinics remained open while other nearby or related clinics were closed, providers mentioned that the resulting increased patient caseload was also a source of stress.

### Changing clinical guidelines and protocols

The need for providers to quickly and continuously adapt to changing clinical guidelines and protocols was another source of stress and anxiety. This included confusion about recommendations and the consequent stress of clinical decision-making. A nurse practitioner said:

“More worry and concern; the unknown. Changes daily. This is going to be the new ‘normal’. Not looking forward to how medicine will be done in future.”

*Nurse Practitioner, Youth/Student Health Center, Oregon*

A physician in leadership explained:

“I work in leadership, and I need to lead very unpopular changes that I myself don’t agree with.”

*Internal Medicine Physician, Primary Care Clinic, Minnesota*

### Telehealth visits

Navigating telehealth was a significant source of stress. Seventy-seven percent of reproductive health providers said they were currently offering telehealth services during the epidemic, with most of them offering a mix of in-person and telehealth visits. Providers expressed feeling stressed due to the challenges of integrating telehealth visits, not being able to provide full patient exams, and feeling that they had limited resources to care for both COVID-19 and non-COVID-19 patients over the phone/video. One provider attributed their stress and anxiety to:

“Fear of missing diagnoses due to telehealth or attention distracted by COVID-19.”

*Physician Assistant, Primary Care Clinic, North Carolina*

### Anxiety about COVID-19 among patients and staff

Another stressor was related to managing both patients’ and other staff’s stress and anxiety about COVID-19. Providers gave examples of “watching patients struggle.” One provider even gave an example of patients becoming more aggressive. Providers also mentioned their own feelings of stress and anxiety related to potentially contracting COVID-19, as well as illness and death among co-workers and patients. In addition to these fears, providers’ need to remain professional while experiencing these situations took a significant toll:

“Responsibility to present a calm and supportive environment to patients and staff causes a repression of your own stress. This is exhausting… I have depleted my emotional reserves calming others.”

*Clinic Manager, Primary Care Clinic, New Mexico*

### Worry about COVID-19 infection and/or infecting family

Providers not surprisingly experienced stress and anxiety related to worries about becoming infected and/or infecting their family. One provider referred to feeling vulnerable because of underlying medical conditions. Other providers described their concerns as follows:

“Always wondering if this is the day that I get the virus.”

*Counselor/Health Educator, Family Planning Clinic, Pennsylvania*

One provider said that, as a result of concern of infecting their family, they were now living apart from their family. Several providers highlighted the lack of PPE as a stressor. Providers also expressed concern about exposure due to asymptomatic patients.

### Financial and employment concerns

Another source of stress and anxiety was due to financial concerns, including job stability, decreased hours and income, as well as potential and actual layoffs for themselves, partners, or other family members. One nurse midwife explained:

“I feel that our administration does not care about patients or quality of care, and that my hard work is not valued in the least. I might be fired like many colleagues at any moment, by text or phone, and I have dedicated my career to serving others through health care, particularly to those marginalized.”

*Certified Nurse Midwife, Primary Care Clinic, New Mexico*

### Home-related concerns including childcare

Another source of stress related to concerns about childcare and the challenges of managing home schooling of children. One provider gave the example of not being able to find childcare, while another described relying on family to provide childcare with accompanying worry about family becoming ill. Another provider said their stress was due to being on duty on all fronts:

“Home all the time working full time and homeschooling 3 children.”

*Family Practice Physician, Primary Care Clinic, California*

### Provider distress and burnout

There were several examples of provider distress, overwhelm, overwork, and burnout. One provider simply said: One provider explained how their work has increased their feelings of stress by simply saying:

“I can’t explain it- it’s horrible.”

*Registered Nurse, Family Planning Clinic, Tennessee*

Providers mentioned not being able to focus and facing decreased motivation to work.

“Feeling overwhelmed, stressed, unsure of what is right or wrong. Don't want to come to work. Feeling down and don't feel like I have support.”

*Nurse Practitioner, School-based Health Center, Oregon*

Several providers mentioned lack of sleep, either due to work or to worrying, as contributing to anxiety:

“I wake up in the middle of the night worrying about COVID.”

*Registered Nurse, Family Planning Clinic, Louisiana*

Several providers mentioned increasing burnout. Providers also referenced isolation as one of the causes of stress and anxiety. A few even related the isolation to their altered working conditions:

“Stuck alone in an office all day with little to no patient interaction.”

*Registered Nurse, Family Planning Clinic, Georgia*

### Fear of the unknown

Finally, another common theme that increased feelings of stress and anxiety related to the fear of the unknown. The unknown was mentioned in relation to COVID-19, uncertainty about when services would re-open, the future of their clinic or organization, and about the virus.

“The unknown always causes anxiety. When will our operations go back to normal? Will there be another spike and will we have to close again?”

*Clinic Manager/Director, Family Planning Clinic, Montana*

## Discussion

This qualitative study offers timely evidence of how providing care during the US COVID-19 epidemic exacerbates provider stress, anxiety, and depression among US reproductive health providers. There is limited evidence focusing on the mental health consequences for outpatient providers, including reproductive health providers, across different practice settings. This study presents national data across different practice settings and provider types showing that even reproductive health providers, not commonly considered to be on the “front lines” of COVID-19 care, are experiencing negative mental health repercussions. There were no differences by age, gender, race/ethnicity, provider type, practice setting or geographic region between those who reported experiencing stress, anxiety or depression and those who did not, indicating that providers of all types are all facing stress and anxiety as a result of the epidemic. Many providers reporting stress, anxiety or depression mentioned changes in job responsibilities, with several examples of providers managing testing sites. Patient care concerns were one of the main sources of provider distress, with worries about the quality of care, follow-up, COVID-19 management, and access to care for non-COVID-19 patients. The constant need to adapt to changing clinical guidelines and telehealth were also frequently mentioned. Providers noted the challenges of coping with co-worker illness, absence, and deaths. Other sources of stress, anxiety and depression centered around inadequate PPE, fear of coming to work and fear of getting sick or getting family members sick. Financial concerns, including apprehension about unemployment, contributed to provider distress. Childcare responsibilities also represented a challenge. Providers also attributed stress, anxiety and depression to feeling to feeling overwhelmed, being unable to focus, getting insufficient sleep. Finally, the fear of the unknown, related to the disease, the clinic, and implications for their communities and the country as a whole, was mentioned as a source of stress and anxiety.

Our study’s findings, in addition to other recent studies [11, 27, 29, 30], point to the urgent need to ensure that providers across the entire health system have the resources and support to cope with the mental health consequences of providing health care during this epidemic. Several perspective articles have raised concerns about the mental health repercussions of COVID-19 on providers [7–10, 31, 32]. Interventions designed for the new realities of care provision during this epidemic should focus on reducing stigma as a barrier to health providers accessing treatment for mental illness [33–35]; identifying early warning signs of anxiety and depression; providing counseling, treatment and psychosocial support [8, 32, 36]; reducing burnout through structural changes to reduce administrative burdens and residency work hour requirements [18, 37]; and increasing pay and job security for health workers at the bottom of the income distribution [38].

The study has limitations. The sample represents a convenience sample of reproductive health providers in outpatient settings and is not generalizable to all providers in the US. Future research would benefit from a nationally representative sample of outpatient reproductive health providers to provide generalizable results for the US. The study relied on open-ended text responses from a survey and did not use an in-depth interview guide which could provide nuanced responses. However, the open-ended text responses allowed for a larger sample size. The survey did not diagnose clinical anxiety or depression among study participants. Due to the survey design, only providers that mentioned having experienced stress, anxiety and/or depression during the pandemic responded to the follow-up open-ended question to describe how stress, anxiety and/or depression had increased. Some providers may have experienced less stress, anxiety and/or depression due to changes during the pandemic, but these are not captured in the response categories.

## Conclusions

The COVID-19 pandemic is resulting in many lives lost, including among health providers [39]. In addition, reproductive health providers are experiencing significant stress, anxiety, and depression as a result of providing care during the US COVID-19 epidemic. These consequences not only have deleterious effects on the personal health of these providers but may also affect the quality of care that they are able to offer at the exact moment when high quality care is most crucial. Policy and programmatic responses are critically needed to address the adverse mental health consequences of providing care during this pandemic, including among reproductive health providers.

## Data Availability

The datasets used and/or analysed during the current study are available from the corresponding author on reasonable request.

## Abbreviations

COVID-19: Severe coronavirus disease 2019
PPE: Personal protective equipment
